# Effects of a pre- and probiotic mixture and an autogenous vaccine on growth performance in newly weaned piglets experimentally challenged with an enterotoxigenic *Escherichia coli* strain

**DOI:** 10.1093/tas/txad030

**Published:** 2023-03-13

**Authors:** Katharina Zeilinger, Anna G Wessels, Wilfried Vahjen, Jürgen Zentek

**Affiliations:** Institute of Animal Nutrition, Department of Veterinary Medicine, Freie Universität Berlin, 14195 Berlin, Germany; Institute of Animal Nutrition, Department of Veterinary Medicine, Freie Universität Berlin, 14195 Berlin, Germany; Institute of Animal Nutrition, Department of Veterinary Medicine, Freie Universität Berlin, 14195 Berlin, Germany; Institute of Animal Nutrition, Department of Veterinary Medicine, Freie Universität Berlin, 14195 Berlin, Germany

**Keywords:** challenge trial, *Escherichia coli*, prebiotic, probiotic, vaccine, weaning piglets

## Abstract

Beneficial effects of pro- and prebiotics in weanling piglets are of great interest in livestock production. Similarly, the use of specific vaccines is of interest as alternative to antibiotics to reduce postweaning performance losses. The aim of this study was the assessment of the effect of a dual-strain probiotic (*Bacillus subtilis* and *Bacillus licheniformis*) and a prebiotic (fructo-oligosaccharides) as well as the additional vaccination with an autogenous inactivated *Escherichia coli* vaccine on the performance of newly weaned piglets after experimental infection with an enterotoxigenic *E. coli.* Forty piglets at the age of 28 d were randomly allotted to one of five groups: nonchallenged control (NC); challenged positive control (PC); challenged and vaccinated (CV); challenged and diet supplemented with pre- and probiotic mix (CM) and challenged, diet supplemented with pro- and prebiotic mix and vaccinated (CMV). Piglets of CV and CMV were vaccinated parenterally prior to the trial at the age of 17 d. Compared to NC, the experimental infection with *E. coli* resulted in a significant reduction of body weight gain in both vaccinated groups (*P* = 0.045), which was associated with an impaired gain to feed ratio (*P* = 0.012), but not feed intake. In contrast, piglets in the group supplemented with pro- and prebiotics (group CM) were able to maintain their weight and had an average daily gain, which was not significantly different from groups NC and PC. No differences regarding body weight gain, feed intake, gain to feed ratio and fecal score were observed between groups during the 3rd and 4th week of the trial. A significant impairment of fecal consistency and frequency of diarrhea was observed related to the oral challenge when comparing PC and NC treatments (*P* = 0.024). Neither vaccine, nor supplementation with pro- and prebiotics were able to significantly improve fecal consistency, nor did they have a positive effect on the prevalence of diarrhea. The results show no positive synergistic effect of the specific combination of vaccine and pre- and probiotics used in this trial on performance and diarrhea. The results show that the concept of a combination of a specific vaccination and a probiotic with a prebiotic needs further investigation. In the sense of avoiding the use of antibiotics, this seems to be an attractive approach.

## INTRODUCTION

Weaning is one of the most critical periods in intensive swine production. Piglets are stressed due to separation from their mother, absence of milk, new environment, grouping and mixing of litters and the supply of solid feed. The interruption of the supply of maternal antibodies with the milk represents a challenge for the immature immune system of young piglets (Bailey et al., 1992; Salak-Johnson and Webb, 2018). The switch from highly digestible milk to less digestible, more complex solid feed is another factor that contributes to reduced or completely discontinued feed intake (FI), which then leads to morphological changes, microbial dysbiosis, and inflammation in the intestine (Lalles et al., 2007). In addition, the digestive tract undergoes numerous physiological, immunological, and microbiological changes during this critical postweaning period (Lalles et al., 2004). These alterations in the digestive tract associated with weaning and infections with enteropathogens can result in a high prevalence of postweaning diarrhea (PWD). Clinical signs of PWD are watery diarrhea associated with inappetence, dehydration, and growth depression. The most common pathogen causing PWD are enterotoxigenic *Escherichia coli* (*E. coli*; ETEC; Fairbrother et al., 2005). ETEC strains produce adhesins and enterotoxins, which are crucial to the development of PWD. The predominant fimbrial adhesins in weaning piglets, which are important for ETEC strains to adhere to the small intestinal epithelial cells, are F4 (45.1%) and F18 (33.9%; Luppi et al., 2016). After colonization, the ETEC strains produce heat labile and heat stable enterotoxins which are responsible for secretion of electrolytes into the intestinal lumen, thus creating a hypertonic environment in the gut. The most common enterotoxins are heat-stable toxin b (STb, 59.1%), heat-stable toxin a (STa, 38.1%) and heat-labile toxin (LT, 31.9%) (Luppi et al., 2016).

Concerns about the promotion of antimicrobial resistance due to selective pressures on bacterial populations led many states to implement measures and regulations in order to limit the use of antibiotics in farm animals. The consequence of this was the ban on antibiotics as growth promoters in the European Union since 2006 (Regulation (EC) No. 1831/2003). Following the ban, other nonantibiotic feed additives were becoming more interesting regarding reduction of ETEC load, improving intestinal health and performance of weaned piglets (Halas et al., 2009). Within this group, probiotics either alone or combined with prebiotics and the use of autogenous vaccines have great potential to become important alternatives in developing antibiotic-free feeding strategies (Hedegaard and Heegaard, 2016).

Probiotics are defined as “live micro-organisms that, when administered in adequate amounts, confer a health benefit on the host” (FAO, 2006). However, the use of probiotics provides contradictory results. Many studies report beneficial effects on piglets health and impact on the gut e.g. with some trials showing increased weight gain and improved feed conversion in piglets as reviewed by Barba-Vidal et al. (2018), while others have not shown any effects especially on growth performance (Kreuzer et al., 2012;Hu et al., 2019). *Bacillus* spp. have a high potential to serve as probiotic feed additives and are therefore widely used in the feed industry (Larsen et al., 2014). It has been demonstrated, that *Bacillus subtilis* and *Bacillus licheniformis* can be used to prevent diarrhea, improve gut barrier function, modify immunity of weaned piglets and beneficially influence the intestinal microbial composition and metabolic activity (Lu et al., 2018; Kim et al., 2019;Wang et al., 2021).

Prebiotic carbohydrates are indigestible to the host and are fermented and utilized by the intestinal microbiota (Gibson and Roberfroid, 1995). They can help to enhance the growth of beneficial bacteria selectively, such as Lactobacilli and Bifidobacteria (Kolida et al., 2002; Zhou et al., 2021) and alter the composition of gut microbiota (Zhou et al., 2021). Fructo-oligosaccharides (FOS) occur naturally in a variety of fruits and vegetables, such as artichokes or chicory, and can stimulate the growth of beneficial bacteria in the intestine. In mice, it is reported that FOS can strengthen the immune function by regulating immune parameters, e.g. increased fecal IgA and decreased production of IL-1β (Delgado et al., 2012). In piglets, FOS supplementation increased relative abundance of *Bifidobacterium* spp. and *Lactobacillus* spp. (Zhao et al., 2019) and improved growth performance due to reduction of diarrhea, improved feed intake (FI), and feed conversion (Xu et al., 2005).

As passive lactogenic protection is rapidly lost after weaning, vaccines can help stimulating the active immunity to prevent *E. coli* induced piglet losses. Farms having problems with recurring ETEC infections are often recommended to vaccinate piglets. Vaccines can be administered orally or parenterally (Melkebeek et al., 2013). Products are commercially available and have been successfully tested in weaned piglets (Melkebeek et al., 2013). It was demonstrated that significant reduction of diarrhea, ileal colonization, and fecal shedding of ETEC F4 in challenged piglets after weaning can be achieved (Fairbrother et al., 2017).

Pre- and Probiotics were selected specifically for the *E. coli* strain used in this trial using a novel established ex vivo assay method (Zeilinger et al., 2021). Thus, the recommendations of the consensus committee on the definition of prebiotics were implemented as far as possible, namely the selection of fermentable carbohydrates acting effectively with the probiotic (Swanson et al., 2020). The concurrent combined use of vaccination and pro- and prebiotics may be a promising approach for the future control of PWD, but has not been studied for this purpose. Therefore, the aim of this study was to investigate whether probiotic and prebiotic-based diets combined with an autogenous parenteral vaccine have a beneficial effect on the growth performance and prevalence of diarrhea of newly weaned piglets challenged with an ETEC strain.

## MATERIALS AND METHODS

The study was approved by the State Office of Health and Social Affairs (Landesamt für Gesundheit und Soziales, Berlin; registration G 0109/20). The institutional and national guidelines for animal welfare were followed.

### Animals and Experimental Design, Vaccine, and Challenge Bacterium

Prior to the trial, piglets were tested for their F4ab/F4ac receptor status. DNA extraction was performed according to the manufacturer protocol (DNeasy Blood & Tissue Kit, QIAGEN). Tissue samples for DNA extraction were obtained from piglets on the 1st day of life during ear tagging. Single-nucleotide polymorphisms (SNPs) genotyping, according to the protocol of Kreuzer et al. (2013) was used to determine the receptor status for F4ab/F4ac fimbriae of a total of 145 piglets. Only F4ab/F4ac sensitive piglets were selected for the trial. For the parenteral vaccination, an inactivated whole-cell vaccine (S20-0171) for pigs specifically developed for the *E. coli* challenge strain was used. The vaccine contained aluminum hydroxide as adjuvant and was used at a dosage of 1 mL/piglet (Ripac Labor GmbH, Potsdam, Germany). Two weeks prior to the infection, 20 F4ab/F4ac sensitive piglets (17 ± 3 d old) were randomly selected and vaccinated. A total of 40 piglets (German Landrace, initial BW of 6.42 ± 0.50 kg, weaned at 28 ± 3 d of age) were randomly allocated (considering the vaccination status) to one of five treatment groups (*N* = 8 animals/treatment): nonchallenged control (NC); challenged positive control (PC); challenged and vaccinated (CV); challenged and diet supplemented with pre- and probiotic mixture (CM) and challenged, diet supplemented with pro- and prebiotic mixture and vaccinated (CMV). Piglets were housed individually and observed over a period of 29 d. Feed and water were provided ad libitum. After a 5 d period of adaptation, piglets belonging to the treatment groups PC, CM, CV, and CMV were orally challenged with 2 mL of an *E. coli* isolate (IMT 203/7, serotype O149:K91, hemolytic; positive for F4, F6, paa, LTI, STI, STII, and EAST toxins) at 3 × 10^9^ cfu/mL. The *E. coli* suspension was prepared on the day of the infection following a standard protocol (Schroeder et al., 2006). In short, a preculture of the strain was incubated overnight at 37 °C with intermediate shaking (~150 rpm) in 10 mL LB medium followed by a second preculture with 8 h incubation. The main culture was cultivated in 150 mL LB medium for 11 h at 37 °C with intermittent shaking. After centrifugation (3,000 × *g* for 15 min at 20 °C), cells were diluted in sterile 1% peptone water and density adjusted to 5 × 10^9^/mL. Syringes were filled with 2 mL cell suspension and orally applied to the piglets. Piglets of group NC received 2 mL sterilized water as placebo. For quality control, the actual cell count of 3 × 10^9^ cfu/mL was determined by serial dilution on LB broth (Lennox) agar plates (Carl Roth GmbH + Co. KG, Germany). During the trial, the piglets were monitored daily including general condition, FI, frequency of respiration, behavior, and presence of pain. Fecal consistency was assessed every morning according to a scoring system ranging from 0 to 2 (0 = normal feces, 0.5 = pasty feces, 1 = soft feces with liquid parts, 1.5 = pasty feces with great liquid parts, 2 = liquid diarrhea). Body weight (BW) and FI were recorded weekly to calculate average daily gain (ADG), average daily feed intake (ADFI), and gain to feed (G:F) for each piglet.

### Experimental Diets, Prebiotic, and Probiotic

A basal diet was formulated to meet the nutrient recommendations for weaning piglets (GfE, 2006). The main ingredients and complete diets were analyzed via proximate analysis: dry matter (DM; Method III 3.1); crude protein (Method III 4.1.1 modified after Makro-N-determination, Vario Max CN); crude fiber (Method III 6.1.4); fat (Method III 5.1.1); ash (Method III 8.1); starch (Method III 7.2.1) (VDLUFA, 2012). Calcium concentration was analyzed after dry ashing using atomic absorption spectrometry (AAS Vario 6, Analytik Jena GmbH, Germany). Phosphorus was measured by the vanadate molybdate method. Sugar content in the diets was analyzed by LUFA Nord-West, Germany. The energy content was calculated using the “Prediction of metabolizable energy of compound feeds for pigs” of the Society for Nutrition (GfE, 2008). The composition and nutritional characteristics of the basal diet are given in Tables 1 and 2. The basal diet was supplemented with either pro- and prebiotics or corn starch according to the treatment group. The treatment group NC, PC, and CV received the basal diet with additional corn starch. The two groups CM and CMV received the basal diet with the pro- and prebiotic. A detailed description of the treatment groups is shown in Table 3. A dual-strain commercial probiotic mixture in powder form containing *B.s licheniformis* DSM 5749 and *B. subtilis* DSM 5750 (3.25 × 10^9^ cfu/g) in a ratio of 1:1 (BioPlus, Biochem, Germany) was used at a dose of 400 g/t feed according to the manufacturer recommendation. Both strains are licensed in the EU as feed additives for piglets. A commercial prebiotic product, produced by partial enzymatic hydrolysis from chicory inulin (Orafti P95, beneo, Belgium) was used as source of FOS.

**Table 1.** Ingredients and analyzed nutrient composition of the basal diet^1^ and basal diet supplemented with pre- and probiotics^2^ (as-fed)

Ingredients, %	
Corn	21.03
Wheat	25.00
Soybean meal	23.30
Rye	15.00
Skim milk powder	10.00
Limestone	1.46
Mineral pre-mixture^3^	1.20
Mono calcium phosphate	1.05
Soya oil	1.00
l-lysine HCI	0.53
dl-methionine	0.19
l-threonine	0.18
l-tryptophan	0.06

^1^Diet for NC, PC and CV supplemented with 1% cornstarch.

^2^Diet for CM and CMV supplemented with 1% Fructo-oligosaccharides and 400 g/t BioPlus.

^3^Contents per kg premix: 400,000 IU Vit. A (acetate); 120,000 IU Vit. D3; 8,000 mg Vit. E (α-Tocopherol acetate); 200 mg Vit. K3 (MSB); 250 mg Vit. B1 (Mononitrate); 420 mg Vit. B2 (cryst. Riboflavin); 2,500 mg Niacin (Niacinamide); 400 mg Vit. B6 (HCl); 2,000 μg Vit. B12; 25,000 μg Biotin (commercial feed grade); 1000 mg pantothenic acid (Ca d-Pantothenate); 100 mg folic acid (commercial feed grade); 80,000 mg choline (chloride); 5,000 mg zinc sulfate; 5,000 mg iron carbonate; 6,000 mg manganese sulfate; 1,000 mg copper sulfate-pentahydrate; 20 mg sodium selenite; 45 mg calcium iodate; 130 g sodium chloride; 55 g Mg magnesium sulfate.

NC, nonchallenged control; PC, challenged positive control; CV, challenged and vaccinated; CM, challenged and diet supplemented with pre- and probiotic mixture and CMV, challenged, diet supplemented with pro- and prebiotic mixture and vaccinated.

**Table 2.** Analyzed nutrient composition of the basal diet^1^ and basal diet supplemented with pre- and probiotics^2^ (as-fed)

	Basel Diet^1^	Supplemented Diet^2^
Analyzed nutrient composition, g/kg (as-fed)		
Dry matter	889.0	893.0
Crude ash	51.5	54.0
Crude protein	189.0	184.0
Crude fiber	23.9	23.7
Crude fat	26.4	26.5
Starch	413.0	400.0
Sugar	6.9	6.9
Calcium	8.3	9.0
Phosphorus	5.8	5.8
Calculated metabolizable energy, MJ/kg	13.9	13.8

^1^Treatment groups NC, PC, CV; diet supplemented with 1 % Cornstarch.

^2^Treatment groups CM, CMV; diet supplemented with 1 % Orafti P95 (beneo, Germany) and 400 g/t BioPlus (Biochem, Germany).

NC, nonchallenged control; PC, challenged positive control; CV, challenged and vaccinated; CM, challenged and diet supplemented with pre- and probiotic mixture; CMV, challenged, diet supplemented with pro- and prebiotic mixture and vaccinated. PC, CV, CM, CMV were challenged with 3 × 10^9^ cfu/mL *E. coli* IMT 203/7.

**Table 3.** Experimental design to test combinations of pre-/probiotics and an autogenous vaccination in an *Escherichia coli* challenge model with piglets

Treatment group	Combined pro-/prebiotic	Vaccination	Challenge with *E. coli*
NC	No	No	No
PC	No	No	Yes
CV	No	Yes	Yes
CM	Yes	No	Yes
CMV	Yes	Yes	Yes

NC, nonchallenged control; PC, challenged positive control; CV, challenged and vaccinated; CM, challenged and diet supplemented with pre- and probiotic mixture; CMV, challenged, diet supplemented with pro- and prebiotic mixture and vaccinated.

Number of piglets for each treatment *N* = 8.

Probiotic: *Bacillus licheniformis* DSM 5749 and *Bacillus subtilis* DSM 5750 (3.25 × 10^9^ cfu/g) in a ratio of 1:1, 400 g/t (BioPlus, Biochem, Germany); Prebiotic: 1% Fructo-oligosaccharides (Orafti P95, beneo, Germany); Vaccine: autogenous vaccine (Ripac Labor GmbH).

Challenge strain: *E. coli* isolate (IMT 203/7, serotype O149:K91, hemolytic, 2 mL infection dose per animal, 2.95E+09 CFU/mL).

### Statistical Analysis

The statistical analyses were performed using the software package SPSS (IBM SPSS Version 25). Zootechnical parameters were analyzed by one-way ANOVA based on treatment groups. Prior to this, all data were tested for normal distribution and variance homogeneity using Shapiro–Wilk test and Levene’s test. Treatment groups were compared to each other either using Tukey test or Games-Howell test depending on variance homogeneity. Daily fecal score was analyzed using the Kruskal–Wallis test. The average number of days with diarrhea (fecal score > 0.5) and days without diarrhea (fecal score = 0) as a metric variable was analyzed by ANOVA and Tukey test. All statistical tests used are noted in the footnotes of the respective data tables. Mean differences with a probability of *P* < 0.05 were accepted as statistically significant.

## RESULTS

### Zootechnical Performance

The effects of the bacterial challenge and the different dietary treatments on BW, ADG, and G:F are presented in Table 4. Piglets in vaccinated groups CV and CMV had significantly lower ADG than NC in the first week after infection (*P* = 0.045).

**Table 4.** Effects of dietary supplementation of a probiotic mixture (*B. subtilis* and *B. licheniformis*) and fructo-oligosaccharides (FOS) and/or an *E. coli* autogenous vaccination on the growth performance of weanling piglets infected with an *E. coli* strain 1 wk after weaning

Parameter	Week	NC	PC	CV	CM	CMV	SEM	*P*-value
Body weight, kg	0	6.9	6.9	7.4	6.5	7.2	0.12	0.214
	1	7.8	7.1	7.0	7.2	6.9	0.19	0.558
	2	9.6	9.2	9.6	9.4	9.0	0.25	0.935
	3	13.9	13.6	14.7	14.0	13.4	0.31	0.795
	4	18.1	16.9	17.9	17.3	17.9	0.37	0.845
Daily gain, g/d	1^2^	125^b^	23^a,b^	−61^a^	96^a,b^	−42^a^	28.7	**0.045**
	2	268	306	382	323	309	31.1	0.873
	3	603	625	723	651	619	23.7	0.612
	4	603	474	460	467	640	38.9	0.450
Average daily gain, g/d	1–4	386	345	363	371	368	12.0	0.879
Feed intake, kg/w	1	1.34	0.99	1.62	1.35	1.13	0.088	0.178
	2	2.95	3.42	2.96	2.87	3.29	0.220	0.928
	3	6.30	5.67	5.94	5.73	5.15	0.303	0.828
	4	6.20	5.54	5.69	5.95	5.83	0.221	0.843
G:F	1^2^	0.61^b^	−0.27^a^	−0.29^a^	0.32^a,b^	−0.42^a^	0.126	**0.012**
	2	0.67	0.63	0.98	0.85	0.64	0.074	0.562
	3	0.69	0.78	0.87	0.75	0.80	0.038	0.602
	4	0.69	0.50	0.57	0.53	0.80	0.046	0.478
Average G:F	1–4	0.69	0.64	0.65	0.67	0.66	0.012	0.639

Piglets were weaned at 28 ± 3 d of age with an initial body weight of 6.42 ± 0.50 kg; Body weight at week 0: Initial weight 1 d before challenge, G:F = gain to feed.

NC, nonchallenged control; PC, challenged positive control; CV, challenged and vaccinated; CM, challenged and diet supplemented with pre- and probiotic mixture; CMV, challenged, diet supplemented with pro- and prebiotic mixture and vaccinated. PC, CV, CM, CMV were infected with 3 × 10^9^ cfu/mL *E. coli* IMT 203/7.

^1^Data are presented as means. Means were compared using ANOVA. Values within a row with different superscripts differ significantly at *P* ≤ 0.05.

^2^Kruskal–Wallis test.

However, the mean BWs of the groups were not significantly different (*P* = 0.558), although weight loss was observed in groups CV and CMV during the first week after the challenge. The NC, PC, and CM groups had positive but similar ADGs during the first week after challenge.

Neither supplementation with pre- and probiotics nor vaccination showed significant effects on piglet growth performance. Final BW was similar among all treatments.

Significant differences in G:F ratio were observed during the first week after infection. Except for CM all treatment groups displayed a reduced G:F ratio (*P* = 0.012) compared to NC. Additional statistical analysis using a 2 × 2 design (with-without pre-/probiotic supplementation × with-without vaccination) were used to analyze main effects of pre- and probiotic, vaccine and their interaction (Supplementary Table S1). Except for one outcome regarding ADFI during week 1 (*P* = 0.028), no significant differences were noticed regarding the variables.

### Fecal consistency and diarrhea

During the first 2 wk postchallenge, the fecal consistency differed significantly among the nonchallenged and the challenged control groups. Treatment groups had a mean fecal score of 0.5 but did not differ significantly from NC (Table 5). PC piglets had the highest number of days with pasty feces, which is represented by a fecal score above 0.5. The difference to the other treatment groups was only numerical (*P* = 0.059). The treatments had no effect on the incidence of diarrhea. An additional presentation of the average fecal scores over the entire experimental period of 4 wk (Table 6) revealed a significant difference between the NC and PC groups during the first week (*P* = 0.009). During the subsequent 3 wk, no differences in the fecal score between groups were detected. Individual fecal scores of all piglets can be found in Supplementary Table S2.

**Table 5.** Effects of dietary supplementation of a probiotic mixture (*B. subtilis* and *B. licheniformis*), a fructo-oligosaccharide (FOS) and an *E. coli* autogenous vaccination on the fecal consistency of weanling piglets during the first 2 weeks postchallenge

Parameter	NC	PC	CV	CM	CMV	SEM	*P*-value
Median	0.18^a^	0.59^b^	0.47^a,b^	0.45^a,b^	0.51^a,b^	0.047	**0.024**
Days with score > 0.5	1.88	5.50	4.20	4.67	4.17	0.445	0.059

Median = average fecal score (0 = normal feces, 0.5 = pasty feces. 1 = soft feces with liquid parts, 1.5 = pasty feces with great liquid parts, 2 = liquid diarrhea).

Days > 0.5 = average number of days with a score above 0.5.

NC, nonchallenged control; PC, challenged positive control; IV, challenged and vaccinated; CM, infected and diet supplemented with pre- and probiotic mixture; CMV, challenged, diet supplemented with pro- and prebiotic mixture and vaccinated. PC, CV, CM, CMV were additionally infected with 3 × 10^9^ cfu/mL *E. coli* IMT 203/7.

The average number of days with diarrhea (fecal score > 0.5) was analyzed by ANOVA and Tukey test, means with different superscripts in a row differ significantly (*P* ≤ 0.05) and fecal score was analyzed using the Kruskal–Wallis test.

**Table 6.** Average weekly fecal scores of treatment groups during the trial period^1^

Parameter	Week	NC	PC	CV	CM	CMV	SEM	*P*
Average fecal score	1^2^	0.11^b^	1.07^a^	0.93^a,b^	0.91^a,b^	0.93^a,b^	0.097	**0.009**
	2	0.26	0.19	0.07	0.30	0.12	0.043	0.398
	3	0.02	0.12	0.03	0.23	0.08	0.032	0.147
	4	0.00	0.00	0.00	0.06	0.00	0.010	0.117

NC: nonchallenged control; PC: challenged positive control; CV: challenged and vaccinated; CM: challenged and diet supplemented with pre- and probiotic mixture and CMV: challenged, diet supplemented with pro- and prebiotic mixture and vaccinated. PC, CV, CM, CMV were additionally challenged with 3 × 10^9^ cfu/mL *E. coli* IMT 203/7.

Fecal score (0 = normal feces, 0.5 = pasty feces, 1 = soft feces with liquid parts, 1.5 = pasty feces with great liquid parts, 2 = liquid diarrhea).

^1^Data presented as means.

^a,b^Values within a row with different superscripts differ significantly at *P* ≤ 0.05.

^2^Kruskal–Wallis for rank variable.

## DISCUSSION

The data from the trial suggest, that the use of an autogenous vaccine had no positive effect on piglet growth performance and diarrhea incidence. Contrary to expectations, the pre- and probiotic combination either alone or in combination with the vaccine also had no significant effect on the clinical symptoms after the *E. coli* infection. Growth of the animals was numerically better than in the vaccinated groups, but was not significantly different from the control groups. Since our experimental approach represents a novel strategy of combining pre- and probiotics and vaccination, comparison with existing literature is difficult. However, many studies dealing with either the use of prebiotics or probiotics or vaccinations show positive effects on weaning piglets, mostly in terms of improving performance (Nadeau et al., 2017;Lu et al., 2018; Kim et al., 2019), reduced incidence of diarrhea (Lin et al., 2013; Fairbrother et al., 2017;Nadeau et al., 2017) or positive impact on microbiota composition regarding diversity and relative abundance of beneficial species (Hu et al., 2019; Ding et al., 2021;Wang et al., 2021). Regarding the pre- and probiotic groups, we had expected a similar effect as in the studies mentioned above. However, the CM group only had a positive effect on the performance parameters during the first week of the trial in direct comparison with the vaccinated groups. In comparison with the control groups, no clear effect was observed. In contrast, vaccination did not appear to have a stabilizing effect on performance parameters, as all animals in the vaccinated groups had significantly reduced growth. The question arises to what extent vaccination actually affected the performance of piglets in the CMV group. Considering these results, it is only speculative to assume that there was an influence. However, due to the lack of further analysis, a direct impact of the vaccine on the health of the piglets remains speculation. An alternative factorial analysis of the data was also unable to demonstrate effects of the treatments. However, a biological effect between the groups is evident, as weight loss during the first week of the experiment was observed in the vaccinated animals. On farms, it matters greatly in terms of animal welfare and profitability whether piglets gain weight after weaning or lose weight due to diarrhea or infection. There is a risk that affected piglets will not recover after the growth retardation and will be severely underdeveloped (Rhouma et al., 2017). If many piglets are affected on a farm, this can lead to major economic losses (Niemi, 2021). In the experiment conducted here, all vaccinated animals showed decreased performance, but recovered during the course of the experiment. Even though the alternative analysis of the data did not show a significant difference here, it is still an interesting fact in the overall assessment. Why the data show a significant difference in daily FI during the first week of the experiment is not clear. However, it is possible that the rather small sample and the high variability of the data led to this result.

Contrary to expectations, the study did not detect beneficial effects of vaccination on the parameters studied. The piglets received one parenteral vaccination around 17 d of age without additional boostering. Piglets are born with a limited immune competence as there is no significant transfer of maternal antibodies in utero (Rooke and Bland, 2002). Passive immunization of piglets occurs up to 48 h after birth through the high concentrations of immunoglobulins in the colostrum. Over the next 2 to 3 d, there is the transition from colostrum to milk (Langel et al., 2016), associated with a decline in immunoglobulins. However, a certain level of immunoglobulins is retained in the milk. A possible influence on the process of active immunization by maternal antibodies cannot be excluded (Snoeck et al., 2003), as the timing of vaccination was relatively early at 17 d. It can be assumed that colostral and milk antibodies deliver a transient passive protection but are rapidly degraded making piglets susceptible to ETEC infection after weaning (Haesebrouck et al., 2004; Hedegaard and Heegaard, 2016; Tizard, 2020). Parenteral vaccination of sows can effectively prevent the occurrence of ETEC diarrhea in neonates until weaning. The dams in this study, however, were not vaccinated against ETEC. Parenteral ETEC vaccines, as used in the current trial, do not always result in a long-term intestinal mucosal IgA response and rather tend to stimulate the systemic immune response (Melkebeek et al., 2013; Matías et al., 2017). In a study conducted by Bianchi et al. (1996), parenterally immunized piglets showed a lack of enteric immune response after subsequent oral infection with *E. coli*. Oral vaccines should be preferred to parenteral vaccines, especially in young animals, not only because of cost effectiveness and adjuvants used, but also because booster vaccinations, if needed, are easier to administer (Dubreuil, 2021). Oral vaccination provides more effective protection for weaned piglets, as demonstrated in several ETEC challenge models, and therefore appears to be a more reliable method of protecting piglets from ETEC infection (Fairbrother et al., 2017; Nadeau et al., 2017). Oral vaccines can be manufactured more cost-effectively, are easy to administer, safe, stable, and suitable for large-scale use (Dubreuil, 2021). In addition to the route of administration, ETEC vaccines differ greatly in their composition. Autogenous vaccines, as used here, are simple to produce and have economic advantages. They have the benefit of presenting a range of complex antigens to the immune system and, unlike live attenuated vaccines, provide a high level of safety (Ramjeet et al., 2008). Ideally, vaccines should contain fully inactivated cells that retain their antigenic properties to provide adequate immunity (Kaminski et al., 2014). An autogenous vaccine should therefore be able to induce a strong immune protection against specific pathogens. However, the efficacy and safety of autogenous vaccines in the field has not been conclusively elucidated (Hoelzer et al., 2018). Autogenous vaccines often provide only partial protection against one serotype and limited cross-protection (Jolie et al., 1995). In addition, it is possible that the antigens may be altered or destroyed by heat, irradiation, or chemical treatments during the manufacture of the vaccine, thereby compromising its efficacy (Haesebrouck et al., 1997). Problems during manufacturing can also lead to reduced efficacy. Pace et al. (1998), found that the main reason for the unacceptable performance of many whole-cell vaccines is that the bacteria used for manufacture have not yet been able to develop the spectrum of required antigens at the time of their inactivation. It is unlikely that a vaccine containing these bacteria can elicit sufficient immunity. Still, the observed reduction in performance after vaccination was unexpected. We assume that the vaccination led to an intensive immune reaction, which expressed itself in a reduced growth performance and increased incidence of diarrhea. The exact reasons for this remain unclear, as the vaccine used in the trial was made specifically for the study and was not previously tested for potential effects on performance or health.

Another promising approach for improving the performance and health of weaning piglets is the use of probiotics. In order to perform this study, probiotic strains were used which have already led to positive results in other studies, e.g., regarding growth or feed conversion. *Bacillus* spp. are probiotic strains that are often used in weaning piglets (Lu et al., 2018; Luise et al., 2019; Wang et al., 2021). The inhibitory activity of the *Bacillus* spp. strains against pathogenic bacteria (e.g., *E. coli*) is species and strain dependent. Strains belonging to the species *B. subtilis* have great potential to inhibit the growth of pathogens in the intestine.

*Bacillus subtilis* showed better probiotic potential in terms of pathogen inhibition, sporulation, production of glycosyl hydrolases and biofilms compared to *B. licheniformis* (Larsen et al., 2014). However, other studies have shown that the combined dietary supplementation with*Bacillus subtilis* and *B. licheniformis* can further enhance the positive effect on the composition of the microbiota by increasing microbial diversity, metabolic activity and intestinal mucosa (Wang et al., 2021). In our study, improved zootechnical performance by dietary supplementation with prebiotics and probiotics was evident only in direct comparison to the two vaccinated groups and the CM group. Comparison with the control groups showed that pre- and probiotics did not result in comparable growth performance and feed conversion. Effects were only visible during the first week after challenge. For the remaining 3 wk of the trial, no significant differences in piglet performance were observed between the treatment groups. Results are in line with trials conducted by Kim et al. (2019) and Luise et al. (2019), as no effects beyond the first week of infection were observed there either.

The question arises whether an alternate use of the pre- and probiotics could have yielded better results. Whether the short-term intake of the supplemented diet only after weaning was sufficient to stabilize the intestinal health of the piglets or to sufficiently improve their protection against infection cannot be conclusively assessed. An equal intake of prebiotics and probiotics cannot be ensured among all piglets as FI after weaning may vary considerably due to nutritional, environmental, and psychological stress (Dong and Pluske, 2007; Lalles et al., 2007). Due to the unique relationship between sow and offspring in terms of microbiota and gut health (Luhrmann et al., 2021), altering the sow’s microbiota with probiotics may be a way to improve gut health and reduce the incidence of PWD in weanling piglets. This approach in combination with an autogenous vaccine should be further investigated in future studies.

To minimize stress to the animals, the trial was designed to be as noninvasive as possible. Retrospectively, additional analyses e.g., collecting blood samples to measure titers of specific antibodies would probably have provided a more profound insight into the immune response triggered by the vaccine. Indirect ELISA is an easy way to monitor specific IgM and IgA levels in serum and observe modifications occurring after immunization and infection. Moreover, an additional booster vaccination shortly before or after weaning might have triggered a stronger immune response to the infection. To realize the practical application of autogenous vaccines and feed additives on pig farms, an inactivated whole cell vaccine was chosen for this study. Since the use of live attenuated vaccines is subject to strict regulations, it makes sense to use inactivated vaccines also in future trials. However, oral use of the vaccine should be favored. With regard to the recent literature, it would have been particularly interesting to investigate whether the administration of the vaccination led to any remarkable alteration of the microbial community compared to the nonvaccinated animals. This approach could be pursued through further research.

Under the specific conditions of the study conducted here, the efficacy of the pre- and probiotic combination was not convincing although recent literature often reports beneficial effects on intestinal health. Vaccination was also not convincing and instead resulted in reduced performance and increased incidence and severity of diarrhea in challenged piglets possibly indicating a strong immune interaction. Accordingly, synergistic effects were not observed either, however, the potential synergistic effect needs to be verified on a broader basis. The application of the vaccine by the oral route is possibly more promising than the parenteral approach tested in this study and also strain-specific characteristics of the infectious pathogen should be taken more into consideration. Therefore, a general conclusion on the efficacy of such combinations cannot be drawn from one single study, as other combinations and an alternative experimental design may affect the intestinal response differently.

## Supplementary Material

txad030_suppl_Supplementary_Material

## References

[CIT0001] Bailey, M., C. J. Clarke, A. D. Wilson, N. A. Williams, and C. R. Stokes. 1992. Depressed potential for interleukin-2 production following early weaning of piglets. Vet. Immunol. Immunopathol. 34:197–207. doi:10.1016/0165-2427(92)90164-l 1455680

[CIT0002] Barba-Vidal, E., S. M. Martin-Orue, and L. Castillejos. 2018. Review: Are we using probiotics correctly in post-weaning piglets? Animal. 12:2489–2498. doi:10.1017/S1751731118000873 29720287

[CIT0003] Bianchi, A. T. J., J. W. Scholten, A. M. vanZijderveld, F. G. vanZijderveld, and B. A. Bokhout. 1996. Parenteral vaccination of mice and piglets with f4(+) escherichia coli suppresses the enteric anti-f4 response upon oral infection. Vaccine 14:199–206. doi:10.1016/0264-410x(95)00192-4 8920700

[CIT0004] Delgado, G. T. C., R. Thome, D. L. Gabriel, W. Tamashiro, and G. M. Pastore. 2012. Yacon (smallanthus sonchifolius)-derived fructooligosaccharides improves the immune parameters in the mouse. Nutr. Res. 32:884–892. doi:10.1016/j.nutres.2012.09.012 23176799

[CIT0005] Ding, H., X. Zhao, C. Ma, Q. Gao, Y. Yin, X. Kong, and J. He. 2021. Dietary supplementation withbacillus subtilisdsm 32315 alters the intestinal microbiota and metabolites in weaned piglets. J. Appl. Microbiol. 130:217–232. doi:10.1111/jam.14767 32628331

[CIT0006] Dong, G. Z., and J. R. Pluske. 2007. The low feed intake in newly-weaned pigs: Problems and possible solutions. Asian-Australas. J. Anim. Sci. 20:440–452. doi:10.5713/ajas.2007.440

[CIT0007] Dubreuil, J. D. 2021. Pig vaccination strategies based on enterotoxigenic escherichia coli toxins. Braz. J. Microbiol. 52:2499–2509. doi:10.1007/s42770-021-00567-3 34244980 PMC8270777

[CIT0008] Fairbrother, J. M., E. Nadeau, L. Belanger, C. L. Tremblay, D. Tremblay, M. Brunelle, R. Wolf, K. Hellmann, and A. Hidalgo. 2017. Immunogenicity and protective efficacy of a single-dose live ­non-pathogenic escherichia coli oral vaccine against f4-positive enterotoxigenic escherichia coli challenge in pigs. Vaccine 35:353–360. doi:10.1016/j.vaccine.2016.11.045 27916413

[CIT0009] Fairbrother, J. M., E. Nadeau, and C. L. Gyles. 2005. Escherichia coli in postweaning diarrhea in pigs: An update on bacterial types, pathogenesis, and prevention strategies. Anim. Health Res. Rev. 6:17–39. doi:10.1079/ahr2005105 16164007

[CIT0010] FAO 2006. Probiotics in food - health and nutrition properties and guidelines for evaluation. In: FAO (ed.) Food and Nutrition Paper. FAO: Rome.

[CIT0011] GfE 2006. Empfehlungen zur energie-und nährstoffversorgung von schweinen. Germany: DLG.

[CIT0012] GfE. 2008. Prediction of metabolisable energy of compound feeds for pigs. Proc. Soc. Nutr. Physiol. 199–204.

[CIT0013] Gibson, G. R., and M. B. Roberfroid. 1995. Dietary modulation of the human colonic microbiota - introducing the concept of prebiotics. J. Nutr. 125:1401–1412. doi:10.1093/jn/125.6.1401 7782892

[CIT0014] Haesebrouck, F., K. Chiers, I. Van Overbeke, and R. Ducatelle. 1997. Actinobacillus pleuropneumoniae infections in pigs: The role of virulence factors in pathogenesis and protection. Vet. Microbiol. 58:239–249. doi:10.1016/s0378-1135(97)00162-4 9453134

[CIT0015] Haesebrouck, F., F. Pasmans, K. Chiers, D. Maes, R. Ducatelle, and A. Decostere. 2004. Efficacy of vaccines against bacterial diseases in swine: What can we expect? Vet. Microbiol. 100:255–268. doi:10.1016/j.vetmic.2004.03.002 15145504

[CIT0016] Halas, D., C. F. Hansen, D. J. Hampson, B. P. Mullan, R. H. Wilson, and J. R. Pluske. 2009. Effect of dietary supplementation with inulin and/or benzoic acid on the incidence and severity of post-weaning diarrhoea in weaner pigs after experimental challenge with enterotoxigenic escherichia coli. Arch. Anim. Nutr. 63:267–280. doi:10.1080/17450390903020414 26967697

[CIT0017] Hedegaard, C. J., and P. M. H. Heegaard. 2016. Passive immunisation, an old idea revisited: Basic principles and application to modern animal production systems. Vet. Immunol. Immunopathol. 174:50–63. doi:10.1016/j.vetimm.2016.04.007 27185263 PMC7127230

[CIT0018] Hoelzer, K., L. Bielke, D. P. Blake, E. Cox, S. M. Cutting, B. Devriendt, E. Erlacher-Vindel, E. Goossens, K. Karaca, S. Lemiere, et al. 2018. Vaccines as alternatives to antibiotics for food producing animals. Part 2: New approaches and potential solutions. Vet. Res. 49:1–15. doi:10.1186/s13567-018-0561-7 PMC6066917 30060759

[CIT0019] Hu, C. J., W. G. Xing, X. H. Liu, X. Z. Zhang, K. Li, J. Liu, B. C. Deng, J. P. Deng, Y. Li, and C. Q. Tan. 2019. Effects of dietary supplementation of probiotic enterococcus faecium on growth performance and gut microbiota in weaned piglets. AMB Express. 9:1–12. doi:10.1186/s13568-019-0755-z 30825022 PMC6397275

[CIT0020] Jolie, R. A. V., M. H. Mulks, and B. J. Thacker. 1995. ­Cross-protection experiments in pigs vaccinated with actinobacillus-pleuropneumoniae subtypes 1a and 1b. Vet. Microbiol. 45:383–391. doi:10.1016/0378-1135(94)00145-m 7483251

[CIT0021] Kaminski, R. W., M. Wu, K. R. Turbyfill, K. Clarkson, B. Tai, A. L. Bourgeois, L. L. Van De Verg, R. I. Walker, and E. V. Oaks. 2014. Development and preclinical evaluation of a trivalent, formalin-inactivated shigella whole-cell vaccine. Clin. Vaccine Immunol. 21:366–382. doi:10.1128/CVI.00683-13 24403527 PMC3957668

[CIT0022] Kim, K., Y. J. He, X. Xiong, A. Ehrlich, X. D. Li, H. Raybould, E. R. Atwill, E. A. Maga, J. Jorgensen, and Y. H. Liu. 2019. Dietary supplementation of bacillus subtilis influenced intestinal health of weaned pigs experimentally infected with a pathogenic *E. coli*. J. Anim. Sci. Biotechnol. 10:1–12. doi:10.1186/s40104-019-0364-3

[CIT0023] Kolida, S., K. Tuohy, and G. R. Gibson. 2002. Prebiotic effects of inulin and oligofructose. Br. J. Nutr. 87:S193–S197. doi:10.1079/BJNBJN/2002537 12088518

[CIT0024] Kreuzer, S., P. Janczyk, J. Assmus, M. F. G. Schmidt, G. A. Brockmann, and K. Nockler. 2012. No beneficial effects evident for enterococcus faecium ncimb 10415 in weaned pigs infected with salmonella enterica serovar typhimurium dt104. Appl. Environ. Microbiol. 78:4816–4825. doi:10.1128/aem.00395-12 22544257 PMC3416376

[CIT0025] Kreuzer, S., M. Reissmann, and G. A. Brockmann. 2013. Gene test to elucidate the etec f4ab/f4ac receptor status in pigs. Vet. Microbiol. 162:293–295. doi:10.1016/j.vetmic.2012.07.049 22917838

[CIT0026] Lalles, J. P., P. Bosi, H. Smidt, and C. R. Stokes. 2007. Nutritional management of gut health in pigs around weaning. Proc. Nutr. Soc. 66:260–268. doi:10.1017/S0029665107005484 17466106

[CIT0027] Lalles, J. P., G. Boudry, C. Favier, N. Le Floc’h, I. Lurona, L. Montagne, I. P. Oswald, S. Pie, C. Piel, and B. Seve. 2004. Gut function and dysfunction in young pigs: Physiology. Anim. Res. 53:301–316. doi:10.1051/animres:2004018

[CIT0028] Langel, S. N., F. C. Paim, K. M. Lager, A. N. Vlasova, and L. J. Saif. 2016. Lactogenic immunity and vaccines for porcine epidemic diarrhea virus (pedv): Historical and current concepts. Virus Res. 226:93–107. doi:10.1016/j.virusres.2016.05.016 27212686 PMC7111331

[CIT0029] Larsen, N., L. Thorsen, E. N. Kpikpi, B. Stuer-Lauridsen, M. D. Cantor, B. Nielsen, E. Brockmann, P. M. Derkx, and L. Jespersen. 2014. Characterization of bacillus spp. Strains for use as probiotic additives in pig feed. Appl. Microbiol. Biotechnol. 98:1105–1118. doi:10.1007/s00253-013-5343-6 24201893

[CIT0030] Lin, J., K. S. Mateo, M. J. Zhao, A. K. Erickson, N. Garcia, D. He, R. A. Moxley, and D. H. Francis. 2013. Protection of piglets against enteric colibacillosis by intranasal immunization with k88ac (f4ac) fimbriae and heat labile enterotoxin of escherichia coli. Vet. Microbiol. 162:731–739. doi:10.1016/j.vetmic.2012.09.025 23089483

[CIT0031] Lu, X. H., M. Zhang, L. Zhao, K. S. Ge, Z. Y. Wang, L. Jun, and F. Z. Ren. 2018. Growth performance and post-weaning diarrhea in piglets fed a diet supplemented with probiotic complexes. J. Microbiol. Biotechnol. 28:1791–1799. doi:10.4014/jmb.1807.07026 30199923

[CIT0032] Luhrmann, A., K. Ovadenko, J. Hellmich, C. Sudendey, V. Belik, J. Zentek, and W. Vahjen. 2021. Characterization of the fecal microbiota of sows and their offspring from german commercial pig farms. PLoS One 16:e0256112. doi:10.1371/journal.pone.0256112 34398927 PMC8367078

[CIT0033] Luise, D., M. Bertocchi, V. Motta, C. Salvarani, P. Bosi, A. Luppi, F. Fanelli, M. Mazzoni, I. Archetti, G. Maiorano, et al. 2019. Bacillus sp. Probiotic supplementation diminish the escherichia coli f4ac infection in susceptible weaned pigs by influencing the intestinal immune response, intestinal microbiota and blood metabolomics. J. Anim. Sci. Biotechnol. 10:1–16. doi:10.1186/s40104-019-0380-3 31528339 PMC6740008

[CIT0034] Luppi, A., M. Gibellini, T. Gin, F. Vangroenweghe, V. Vandenbroucke, R. Bauerfeind, P. Bonilauri, G. Labarque, and A. Hidalgo. 2016. Prevalence of virulence factors in enterotoxigenic escherichia coli isolated from pigs with post-weaning diarrhoea in europe. Porcine Health Manag. 2:6. doi:10.1186/s40813-016-0039-9 28405446 PMC5382528

[CIT0035] Matías, J., M. Berzosa, Y. Pastor, J. M. Irache, and C. Gamazo. 2017. Maternal vaccination. Immunization of sows during pregnancy against etec infections. Vaccines 5:8. doi:10.3390/vaccines5040048 29211052 PMC5748614

[CIT0036] Melkebeek, V., B. M. Goddeeris, and E. Cox. 2013. Etec vaccination in pigs. Vet. Immunol. Immunopathol. 152:37–42. doi:10.1016/j.vetimm.2012.09.024 23068270

[CIT0037] Nadeau, E., J. M. Fairbrother, J. Zentek, L. Belanger, D. Tremblay, C. L. Tremblay, I. Rohe, W. Vahjen, M. Brunelle, K. Hellmann, et al. 2017. Efficacy of a single oral dose of a live bivalent e-coli vaccine against post-weaning diarrhea due to f4 and f18-positive enterotoxigenic e-coli. Vet. J. 226:32–39. doi:10.1016/j.tvjl.2017.07.004 28911838

[CIT0038] Niemi, J. K. 2021. Advancements and technologies in pig and poultry bacterial disease control. The Economic Cost of Bacterial Infection. Elsevier Academic Press, 2021, p. 1–23.

[CIT0039] Pace, J. L., H. A. Rossi, V. M. Esposito, S. M. Frey, K. D. Tucker, and R. I. Walker. 1998. Inactivated whole-cell bacterial vaccines: Current status and novel strategies. Vaccine 16:1563–1574. doi:10.1016/s0264-410x(98)00046-2 9711805

[CIT0040] Ramjeet, M., V. Deslandes, J. Gouré, and M. Jacques. 2008. Actinobacillus pleuropneumoniae vaccines: From bacterins to new insights into vaccination strategies. Anim. Health Res. Rev. 9:25–45. doi:10.1017/S1466252307001338 18346296

[CIT0041] Rhouma, M., J. M. Fairbrother, F. Beaudry, and A. Letellier. 2017. Post weaning diarrhea in pigs: Risk factors and non-colistin-based control strategies. Acta Vet. Scand. 59:1–19. doi:10.1186/s13028-017-0299-7 28526080 PMC5437690

[CIT0042] Rooke, J. A., and I. M. Bland. 2002. The acquisition of passive immunity in the new-born piglet. Livest. Prod. Sci. 78:13–23. doi:10.1016/s0301-6226(02)00182-3

[CIT0043] Salak-Johnson, S. R., and J. L. Webb. 2018. Short- and long-term effects of weaning age on pig innate immune status. Open J. Anim. Sci. 8:137–150. doi:10.4236/ojas.2018.82010

[CIT0044] Schroeder, B., S. Duncker, S. Barth, R. Bauerfeind, A. D. Gruber, S. Deppenmeier & G. Breves. 2006. Preventive effects of the probiotic escherichia coli strain nissle 1917 on acute secretory diarrhea in a pig model of intestinal infection. Digestive Diseases Sci. 51:724-731. doi:10.1007/s10620-006-3198-8 16614995

[CIT0045] Snoeck, V., N. Huyghebaert, E. Cox, A. Vermeire, S. Vancaeneghem, J. P. Remon, and B. M. Goddeeris. 2003. Enteric-coated pellets of f4 fimbriae for oral vaccination of suckling piglets against enterotoxigenic escherichia coli infections. Vet. Immunol. Immunopathol. 96:219–227. doi:10.1016/j.vetimm.2003.08.003 14592734

[CIT0046] Swanson, K. S., G. R. Gibson, R. Hutkins, R. A. Reimer, G. Reid, K. Verbeke, K. P. Scott, H. D. Holscher, M. B. Azad, N. M. Delzenne, et al. 2020. The international scientific association for probiotics and prebiotics (isapp) consensus statement on the definition and scope of synbiotics. Nat. Rev. Gastroenterol. Hepatol. 17:687–701. doi:10.1038/s41575-020-0344-2 32826966 PMC7581511

[CIT0047] Tizard, I. R. 2020. Porcine vaccines. Vaccines for veterinarians. Elsevier. Elsevier Public Health Emergency Collection: 225–242.

[CIT0048] VDLUFA 2012. Handbuch der landwirtschaftlichen versuchs-und untersuchungsmethodik (vdlufa-methodenbuch), bd. Iii. Die chemische untersuchung von futtermitteln. VerlagDarmstadt (Germany): VDLUFA.

[CIT0049] Wang, X., Z. Tian, M. A. K. Azad, W. Zhang, F. Blachier, Z. Wang, and X. Kong. 2021. Dietary supplementation withbacillusmixture modifies the intestinal ecosystem of weaned piglets in an overall beneficial way. J. Appl. Microbiol. 130:233–246. doi:10.1111/jam.14782 32654235

[CIT0050] Xu, C. L., X. D. Chen, C. Ji, Q. G. Ma, and K. Hao. 2005. Study of the application of fructooligosaccharides in piglets. Asian-Australas. J. Anim. Sci. 18:1011–1016. doi:10.5713/ajas.2005.1011

[CIT0051] Zeilinger, K., J. Hellmich, J. Zentek, and W. Vahjen. 2021. Novel ex vivo screening assay to preselect farm specific pre- and probiotics in pigs. Benefe. Microbes 12:567–581. doi:10.3920/bm2020.0226 34420495

[CIT0052] Zhao, W. S., M. Yuan, P. C. Li, H. L. Yan, H. F. Zhang, and J. B. Liu. 2019. Short-chain fructo-oligosaccharides enhances intestinal barrier function by attenuating mucosa inflammation and altering colonic microbiota composition of weaning piglets. Ital. J. Anim. Sci. 18:976–986. doi:10.1080/1828051x.2019.1612286

[CIT0053] Zhou, H., B. Yu, H. Chen, and D. W. Chen. 2021. Carbohydrates effects on nutrition and health functions in pigs. Anim. Sci. J. 92:e13557. doi:10.1111/asj.13557 33899995

