# Supplementary material for: Effects of a pre- and probiotic mixture and an autogenous vaccine on growth performance in newly weaned piglets experimentally challenged with an enterotoxigenic Escherichia coli strain
Source: Transl Anim Sci. 2023 Mar 13;7(1):txad030. doi: 10.1093/tas/txad030 (PMC10037424; doi:10.1093/tas/txad030)
Supplement: txad030_suppl_Supplementary_Material [file txad030_suppl_supplementary_material.docx]

Table S1: Presentation of performance data showing main effects of pre- and probiotic treatment, vaccination and their interaction^1^

| Parameter | Week | Challenged treatment groups | | | | SEM | p-value | | |
| --- | --- | --- | --- | --- | --- | --- | --- | --- | --- |
|  |  | **PC** | **CV** | **CM** | **CMV** |  | **V^2^** | **M^3^** | **I^4^** |
| Body Weight, kg | 1  2  3  4 | 7.1  9.2  13.6  16.9 | 7.0  9.6  14.7  17.9 | 7.2  9.4  14.0  17.3 | 6.9  9.0  13.4  17.9 | 0.19  0.25  0.31  0.37 | 0.675  0.976  0.732  0.348 | 0.967  0.766  0.536  0.859 | 0.859  0.501  0.246  0.809 |
| Daily Gain, g/d | 1  2  3  4 | 23  306  625  474 | -61  382  723  460 | 96  323  651  467 | -42  309  619  640 | 28.7  31.1  23.7  38.9 | 0.163  0.713  0.598  0.449 | 0.530  0.740  0.533  0.409 | 0.712  0.593  0.306  0.371 |
| Average Daily Gain, g/d | 1-4 | 345 | 363 | 371 | 368 | 12.0 | 0.780 | 0.590 | 0.713 |
| Daily Feed Intake, g/d | 1  2  3  4 | 142  489  810  792 | 231  423  848  813 | 193  410  819  850 | 162  470  736  833 | 12.6  31.4  43.3  31.6 | 0.400  0.968  0.879  0.977 | 0.642  0.831  0.605  0.636 | **0.028**  0.394  0.549  0.816 |
| Average Daily Feed Intake, g | 1-4 | 539 | 559 | 549 | 553 | 15.4 | 0.948 | 0.731 | 0.645 |
| G:F | 1  2  3  4 | -0.27  0.63  0.78  0.50 | -0.29  0.98  0.87  0.57 | 0.32  0.85  0.75  0.53 | -0.42  0.64  0.80  0.80 | 0.126  0.074  0.038  0.046 | 0.057  0.844  0.342  0.201 | 0.834  0.405  0.706  0.345 | 0.519  0.364  0.641  0.744 |
| Average G:F | 1-4 | 0.64 | 0.65 | 0.67 | 0.66 | 0.012 | 0.831 | 0.772 | 0.492 |

G:F = gain to feed; PC: challenged positive control; CV: challenged and vaccinated; CM: challenged and diet supplemented with pre- and probiotic mixture and CMV: challenged, diet supplemented with pro- and prebiotic mixture and vaccinated. PC, CV, CM, CMV were challenged with 3x10^9^ cfu/mL E. coli IMT 203/7.

^1^Data are presented as means. Means were compared using MANOVA. Effects of ^2^vaccination, ^3^pre-/probiotic supplementation and ^4^ interaction between vaccination and pre-/probiotic suppl.

*Table S2: Individual fecal score of all piglets used in the trial and involved until the end of the trial.*

|  | **1^1^** | **2** | **3** | **4** | **5** | **6** | **7** | **8** | **9** | **10** | **11** | **12** | **13** | **14** | **15** | **16** | **17** | **18** | **19** | **20** | **21** | **22** | **23** | **24** | **25** | **26** | **27** | **28** | **29** |
| --- | --- | --- | --- | --- | --- | --- | --- | --- | --- | --- | --- | --- | --- | --- | --- | --- | --- | --- | --- | --- | --- | --- | --- | --- | --- | --- | --- | --- | --- |
| **NC1^2^** | 0 | 0 | 0 | 0 | 0 | 0 | 0 | 0 | 0 | 0 | 0 | 2 | 1 | 1 | 1 | 0 | 0 | 0 | 0 | 0 | 0 | 0 | 0 | 0 | 0 | 0 | 0 | 0 | 0 |
| **NC2** | 0 | 0 | 1,5 | 0 | 0 | 0 | 0 | 0,5 | 1 | 0 | 0 | 0 | 0 | 0 | 0 | 0 | 0 | 0 | 0 | 0 | 0 | 0 | 0 | 0 | 0 | 0 | 0 | 0 | 0 |
| **NC3** | 0 | 0 | 1 | 0 | 0 | 0 | 0 | 1 | 1 | 0,5 | 0 | 0 | 0 | 0 | 0 | 0 | 0 | 0 | 0 | 0 | 0 | 0 | 0 | 0 | 0 | 0 | 0 | 0 | 0 |
| **NC4** | 0 | 0 | 0 | 0,5 | 0 | 0 | 0,5 | 0 | 0 | 0,5 | 0 | 0 | 0 | 0 | 0 | 0 | 0 | 0 | 0 | 0 | 0 | 0 | 0 | 0 | 0 | 0 | 0 | 0 | 0 |
| **NC5** | 0 | 0 | 1 | 1 | 0 | 0 | 0 | 0 | 0 | 0 | 1 | 0 | 0,5 | 1 | 0 | 0 | 0 | 0 | 0 | 0 | 0 | 0 | 0 | 0 | 0 | 0 | 0 | 0 | 0 |
| **NC6** | 0 | 0 | 0 | 0 | 0 | 0 | 0 | 0 | 0 | 0 | 0 | 0 | 0 | 0 | 0 | 0 | 0 | 0 | 0 | 0 | 0 | 0 | 0 | 0 | 0 | 0 | 0 | 0 | 0 |
| **NC7** | 0,5 | 0 | 0 | 0 | 0 | 0 | 0 | 0 | 0 | 0 | 0,5 | 0 | 0,5 | 0 | 0 | 0 | 0 | 0 | 0 | 0 | 0 | 0 | 0 | 0 | 0 | 0 | 0 | 0 | 0 |
| **NC8** | 0 | 0 | 0 | 0 | 0 | 0 | 0 | 0 | 0 | 0,5 | 1 | 1 | 0 | 0 | 0 | 0 | 0 | 0 | 0 | 0 | 0 | 0 | 0 | 0 | 0 | 0 | 0 | 0 | 0 |
| **PC1** | 1 | 2 | 2 | 2 | 2 | 2 | 0 | 0 | 0 | 0 | 0 | 0 | 0 | 0 | 0 | 0 | 0 | 0 | 0 | 0 | 0 | 0 | 0 | 0 | 0 | 0 | 0 | 0 | 0 |
| **PC2** | 1 | 0 | 1 | 2 | 0,5 | 0 | 0 | 0 | 0 | 0 | 1 | 0 | 0 | 0 | 0 | 0 | 0 | 0 | 0 | 0 | 0 | 0 | 0 | 0 | 0 | 0 | 0 | 0 | 0 |
| **PC3** | 0 | 1,5 | 2 | 2 | 0 | 1 | 1 | 0 | 0 | 0 | 0 | 0 | 0 | 0 | 0 | 0 | 0 | 0 | 0 | 0 | 0 | 0 | 0 | 0 | 0 | 0 | 0 | 0 | 0 |
| **PC4** | 1 | 2 | 1 | 0 | 0,5 | 0 | 0 | 0 | 0 | 0 | 0 | 0 | 0 | 0 | 0 | 0 | 0 | 1 | 0 | 0 | 0 | 0 | 0 | 0 | 0 | 0 | 0 | 0 | 0 |
| **PC5** | 1 | 2 | 2 | 0,5 | 2 | 2 | 1 | 1 | 1 | 1 | 0,5 | 0 | 0 | 0 | 0 | 0 | 0 | 0 | 0 | 0 | 0 | 0 | 0 | 0 | 0 | 0 | 0 | 0 | 0 |
| **PC6** | 0 | 0 | 2 | 2 | 2 | 1 | 0 | 1 | 0 | 0,5 | 1 | 0,5 | 0,5 | 0 | 0 | 1 | 1 | 1 | 1 | 0 | 0 | 0 | 0 | 0 | 0 | 0 | 0 | 0 | 0 |
| **CV1** | 0,5 | 2 | 1 | 2 | 1,5 | 1 | 0 | 0 | 1 | 1 | 0 | 0 | 0 | 0 | 0 | 0 | 0 | 0 | 0 | 0 | 0 | 0 | 0 | 0 | 0 | 0 | 0 | 0 | 0 |
| **CV2** | 0,5 | 0 | 2 | 2 | 2 | 0 | 0 | 0 | 0 | 0 | 0 | 0 | 0 | 0 | 0 | 0 | 0 | 0 | 0 | 0 | 0 | 0 | 0 | 0 | 0 | 0 | 0 | 0 | 0 |
| **CV3** | 1 | 2 | 2 | 2 | 0 | 0,5 | 0 | 0 | 0 | 0 | 0 | 0 | 0 | 0 | 0 | 0 | 0 | 0 | 0 | 0 | 0 | 0 | 0 | 0 | 0 | 0 | 0 | 0 | 0 |
| **CV4** | 1 | 0 | 2 | 2 | 2 | 1 | 0 | 0 | 0 | 0 | 0 | 0 | 0 | 0 | 0 | 0 | 0 | 0 | 0 | 0 | 0 | 0 | 0 | 0 | 0 | 0 | 0 | 0 | 0 |
| **CV5** | 1 | 1 | 0 | 0 | 0,5 | 0 | 0 | 0 | 0 | 0,5 | 0 | 0 | 0 | 0 | 0 | 0 | 0 | 1 | 0 | 0 | 0 | 0 | 0 | 0 | 0 | 0 | 0 | 0 | 0 |
| **CM1** | 1 | 0 | 0 | 0 | 0 | 0 | 0 | 0 | 0 | 0,5 | 1 | 0 | 0 | 0 | 0 | 0 | 0 | 0,5 | 0 | 0 | 1 | 0 | 0 | 0 | 0 | 0 | 0 | 0 | 0 |
| **CM2** | 2 | 2 | 2 | 1,5 | 2 | 1 | 0 | 1 | 0,5 | 1 | 1,5 | 1 | 1 | 2 | 1 | 1 | 1 | 0,5 | 0 | 0 | 1 | 0 | 0 | 0 | 0 | 0 | 0 | 0 | 0 |
| **CM3** | 2 | 0 | 0 | 2 | 2 | 0 | 0 | 0 | 0 | 0 | 1 | 1 | 1 | 0 | 0 | 0 | 0 | 0 | 0,5 | 0 | 0 | 0 | 0 | 0 | 0 | 0 | 0 | 0 | 0 |
| **CM4** | 1 | 1 | 1 | 1 | 1 | 0 | 0 | 0 | 0 | 0 | 0 | 0 | 0 | 0 | 0 | 0 | 1 | 0 | 0 | 0 | 0 | 0 | 0 | 0 | 1 | 1 | 0 | 0 | 0 |
| **CM5** | 1 | 2 | 2 | 2 | 2 | 1 | 1 | 0 | 0 | 1 | 0,5 | 0 | 0 | 0 | 0 | 0 | 0 | 0 | 0 | 0 | 0 | 0 | 0 | 0 | 1 | 0 | 0 | 0 | 0 |
| **CM6** | 2 | 2 | 2 | 1,5 | 1 | 1 | 1 | 0,5 | 0 | 0 | 0 | 0 | 0 | 0 | 0 | 0 | 0 | 0 | 0 | 0 | 0 | 0 | 0 | 0 | 0 | 0 | 0 | 0 | 0 |
| **CM7** | 0 | 0 | 0,5 | 0 | 0 | 0 | 0 | 0 | 0 | 0 | 0 | 0 | 0 | 0 | 0 | 0 | 1 | 0,5 | 1 | 1 | 0 | 0 | 0 | 0 | 0 | 0 | 0 | 0 | 0 |
| **CMV1** | 1,5 | 1 | 2 | 2 | 2 | 0 | 0 | 0,5 | 0 | 0 | 0 | 0,5 | 0,5 | 0 | 0 | 0 | 0 | 0 | 0 | 0 | 0 | 0 | 0 | 0 | 0 | 0 | 0 | 0 | 0 |
| **CMV2** | 1 | 2 | 2 | 2 | 2 | 0 | 0 | 0,5 | 1 | 0 | 0 | 0 | 0 | 0 | 2 | 0 | 0 | 0 | 0 | 1 | 0 | 0 | 0 | 0 | 0 | 0 | 0 | 0 | 0 |
| **CMV3** | 1 | 0 | 2 | 2 | 2 | 0,5 | 0 | 0 | 0 | 0 | 0 | 0 | 0 | 0 | 0 | 0 | 0 | 0 | 0 | 0 | 0 | 0 | 0 | 0 | 0 | 0 | 0 | 0 | 0 |
| **CMV4** | 0 | 0 | 1 | 2 | 1,5 | 1 | 0 | 0 | 0,5 | 0,5 | 0 | 0 | 0 | 0 | 0 | 0 | 0 | 0 | 0 | 0 | 0 | 0 | 0 | 0 | 0 | 0 | 0 | 0 | 0 |
| **CMV5** | 0 | 0,5 | 0 | 0 | 0 | 0 | 0 | 0 | 0 | 0,5 | 0 | 0 | 0 | 0 | 0 | 0 | 0 | 0 | 0 | 0 | 0 | 0 | 0 | 0 | 0 | 0 | 0 | 0 | 0 |
| **CMV6** | 0 | 1 | 2 | 2 | 2 | 1 | 0 | 0 | 0 | 0,5 | 0 | 0 | 0 | 0 | 0 | 0 | 0 | 0,5 | 0 | 0 | 0 | 0 | 0 | 0 | 0 | 0 | 0 | 0 | 0 |

*^1^ duration of the trial (days); ^2^ treatment groups: NC: non-challenged control; PC: challenged positive control; CV: challenged and vaccinated; CM: challenged and diet supplemented with pre- and probiotic mixture and CMV: challenged, diet supplemented with pro- and prebiotic mixture and vaccinated; Treatment+no. = individual animal; fecal score (0=normal feces, 0,5=pasty feces. 1=soft feces with liquid parts, 1,5= pasty feces with great liquid parts, 2= liquid diarrhea)*
